# Accelerated Design for Perovskite-Oxide-Based Photocatalysts Using Machine Learning Techniques

**DOI:** 10.3390/ma17123026

**Published:** 2024-06-20

**Authors:** Xiuyun Zhai, Mingtong Chen

**Affiliations:** 1 College of Intelligent Manufacturing, Hunan University of Science and Engineering, Yongzhou 425199, China; 2 Public Experimental Teaching Center, Panzhihua University, Panzhihua 617000, China

**Keywords:** photocatalyst, machine learning, ABO_3_-type perovskites, specific surface area, prediction

## Abstract

The rapid discovery of photocatalysts with desired performance among tens of thousands of potential perovskites represents a significant advancement. To expedite the design of perovskite-oxide-based photocatalysts, we developed a model of ABO_3_-type perovskites using machine learning methods based on atomic and experimental parameters. This model can be used to predict specific surface area (SSA), a key parameter closely associated with photocatalytic activity. The model construction involved several steps, including data collection, feature selection, model construction, web-service development, virtual screening and mechanism elucidation. Statistical analysis revealed that the support vector regression model achieved a correlation coefficient of 0.9462 for the training set and 0.8786 for the leave-one-out cross-validation. The potential perovskites with higher SSA than the highest SSA observed in the existing dataset were identified using the model and our computation platform. We also developed a webserver of the model, freely accessible to users. The methodologies outlined in this study not only facilitate the discovery of new perovskites but also enable exploration of the correlations between the perovskite properties and the physicochemical features. These findings provide valuable insights for further research and applications of perovskites using machine learning techniques.

## 1. Introduction

**Figure 1:**
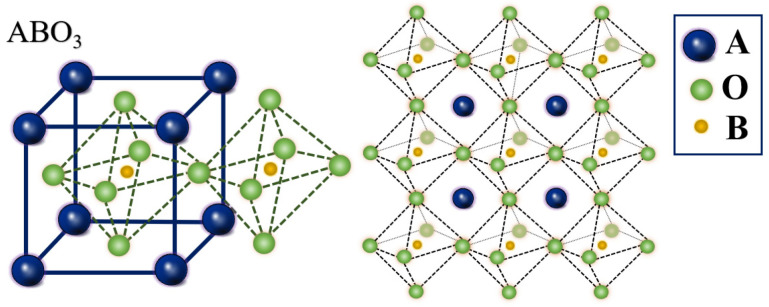
A schematic illustration of the crystal cell of a typical ABO_3_-type perovskite.

Perovskite oxides, with the chemical formula ABO_3_, are a broad category of compounds with similar structures [1,2,3,4]. The earliest compound in this group of compounds is calcium titanate (CaTiO_3_), which was discovered in calcium titanium ore [5]. In perovskite materials, the B-site typically consists of cations with smaller ionic radii. These cations co-ordinate with oxygen anions and occupy the corners of the octahedral structure in the cubic lattice [4,6]. The typical structure of ABO_3_-type perovskites has Pm3m symmetry (Figure 1). Perovskite structures offer significant flexibility for exploration due to the potential replacement of ions at various positions with those of other elements or groups with similar radii [7,8]. This versality results in a diverse array of perovskite compounds, offering a vast potential for specific applications [9,10]. Considering element doping, the potential number of perovskite compounds can reach approximately 10^7^ [11]. These perovskites exhibit a remarkable diversity, offering several superior properties, including high light absorption coefficients [12,13], suitable and adjustable bandgap [14], narrow emission bandwidth [15], long carrier diffusion lengths [16,17], high carrier mobilities [18,19], and cost-effective solution processability [20]. These attributes make perovskites highly promising materials in various fields such as solar cells [21,22], photocatalytic materials [7,23], and photodetectors [24].

Conventional methods of material development typically rely on repeated trial-and-error processes until the desired material properties are achieved [25,26]. Although first principle calculation of materials can provide key characteristics of materials without the need for experimental synthesis [27,28], it involves extensive equation calculations and approximations. However, both experimental methods and first principles calculations face challenges in meeting the demand for large-scale, rapid, and efficient prediction within the vast chemical search space and complex crystal component structures of perovskite-type materials.

Machine learning (ML), as crucial branch of artificial intelligence, can rapidly and effectively evaluate or predict research objects [29,30,31,32]. This highlights the significant potential of ML in the design, synthesis, physical property exploration, and application research of perovskite-type materials [33,34]. ML has been successfully used to accelerate the development of perovskite-oxide-based photocatalysts and solar cell materials in recent years [35,36,37]. ML applications in ABO_3_-type perovskites primarily include predictions related to stability, bandgap, crystal structure, and formability (Table S1 of the Supporting Information). As Experimental data on perovskite synthesis continue to grow, ML is anticipated to play an increasingly vital role in the advancement of perovskite-type photocatalysts [38,39]. 

ABO_3_-type perovskite compounds are a novel class of semiconductor photocatalysts [40], where specific surface area (SSA) is an important indicator of photocatalytic activity [41]. A larger SSA enhances the availability of active sites, thereby improving photocatalytic performance [42]. In this study, a model of ABO_3_-type perovskites for designing perovskite-type photocatalysts with desired SSA is proposed. The model was developed using atomic and experimental parameters with ML methods. The method involved several steps, including data collection, feature engineering, model construction, web-service development, virtual screening, and mechanism elucidation.

ABO_3_-type perovskites are highly promising semiconductor photocatalysts, drawing increasing interest from researchers [43,44]. This study focused on rapidly screening promising ABO_3_-type perovskites for photocatalytic applications and accelerating the design of perovskite-oxide-based photocatalysts. The main contributions of our work are outlined as follows: (1) we compiled a dataset of ABO_3_-type perovskites by gathering experimental data from published references. (2) We developed a support vector regression (SVR) model for synthesizing ABO_3_-type perovskites using the sol–gel method. The model demonstrates high accuracy and good generalization, with correlation coefficients (R) of 0.9462 for the training set and 0.8786 for leave-one-out cross-validation (LOOCV). (3) Key factors influencing the SSA of ABO_3_-type perovskites were identified using forward and backward selection methods based on the SVR model with radial basis function (RBF). (4) The model identified promising ABO_3_-type perovskites with potentially high SSA. (5) An online web service was developed to facilitate rapid and effective prediction of SSA for ABO_3_-type perovskites. This service is accessible at http://1.14.49.110/online_predict/Perovskite (accessed on 7 June 2024).

The other sections of this paper are organized as follows. In Section 2, the main steps involved in constructing the SSA model of ABO_3_-type perovskites are presented in detail, along with the computational details. Feature selection, model construction, model application, and mechanism exploration are presented in Section 3. Section 4 presents the conclusions of the study.

## 2. Material and Methods

### 2.1. Perovskite Model Framework

**Figure 2:**
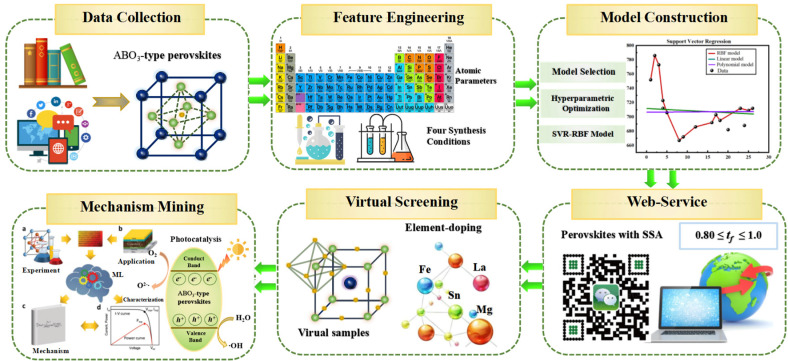
An illustration of the six steps involved in the construction of the model.

The SSA model for ABO_3_-type perovskites was developed through six primary steps, as shown in Figure 2. These steps include data collection, feature engineering, model construction, web-service development, virtual screening, and mechanism mining. The details of each step are as follows.

#### 2.1.1. Data Collection

Data quality is crucial for ML model quality and fundamentally determines its reliability [45]. In most ML research, the upper limit of the model performance depends on the quality of data and features selected, whereas the choice of models and algorithms can only help approach this upper limit infinitely [46]. In this study, we established a reliable dataset by collecting extensive data of ABO_3_-type perovskites. Specifically, we compiled data from 99 perovskite samples sourced from the experimental results reported in the previous literature (Table S2). These ABO_3_-type perovskites were synthesized using the sol–gel technique [47], which involves transforming inorganic compounds or metal alkoxides into oxides or other solid compounds through solidification process involving solution, sol, gel, and subsequent heat-treat treatment. The three values (namely 1, 2, and 3) in the ***PM*** (synthetic mode) column in Table S2 represent three modes of the sol–gel technique: traditional, auto-combustion, and citrate methods. The target variable of the dataset, SSA, denotes the surface area per unit mass of perovskite crystals, measured in square meters per gram (m^2^g^−1^).

The dataset comprised 25 features as the inputs of the ML model (Table S3), including the 4 synthesis conditions (calcination temperature (***CT***), calcination time (***AH***), drying temperature (***DT***), and ***PM***) and 21 atomic parameters. The atomic parameters were derived using the molecular formula of the samples through the Online Computational Platform of Material Data Mining (http://materials-data-mining.com/ocpmdm/) (OCPMDM) [48,49]. The dataset was divided into two parts: the training set comprising 85 samples for constructing the model and the testing set comprising 14 samples (marked with asterisks in Table S2) for external validation. The following rules were observed when dividing the dataset:

(1) The SSA values of the samples in the testing set were kept within the range of values observed in the training set to prevent out of range predictions.

(2) Samples in the dataset were sorted based on the SSA values. Samples in the testing set were selected at regular intervals from this sorted dataset to avoid biased predictions.

In addition, a validation set was established to further verify the model. The validation set comprised three samples (marked with hashes in Table S2) obtained from recently published papers.

#### 2.1.2. Feature Engineering

Ensuring the stability and generalization of the prediction model ensures managing high linear correlations between features used in modeling. An important step in this process involves removing features with strong correlations to build an effective model. To achieve this, Pearson correlation coefficients between the 25 features were calculated, and the correlation matrix is presented in Figure S1. Maximum-relevance minimum-redundancy (mRMR) scores of all features were calculated (Figure S2). Features with lower mRMR scores were eliminated from the dataset if their correlation coefficient with another feature was greater than 0.95. Twenty-three features were retained through this correlative analysis. Features such as the ratio of ionic radius (***R_A_/R_B_***) and unit cell lattice edge (***α***) were eliminated due to their correlation with other features. A feature selection method based on SVR was used to identify the most representative subset from the feature pool. This approach ensures that the selected subset contains essential information with minimal redundancy.

#### 2.1.3. Model Construction

The ML model was designed to establish the relationship between SSA of ABO_3_-type perovskites and the corresponding features. An SVR model with RBF kernel was chosen to accelerate the design of perovskite-type materials. The hyperparameters of the model were optimized using the grid search technique and cross-validation to enhance the prediction performance of the model. The external testing set comprising 14 samples, LOOCV, and an independent validation set were utilized to test the reliability of the model. 

#### 2.1.4. Webserver Development

A webserver refers to software modules operating over a network. It is service-oriented based on distributed programs, allowing users to access data from different web terminals across different locations. Implementing webservers based on the model simplifies the prediction tasks for the clients. Scientists researching perovskite-type materials can use online predictions through the webserver without mastering the intricacies of the ML model.

#### 2.1.5. Virtual Screening

The general molecular formula of ABO_3_-type perovskites is A1*_y_*(A2*_n_*A3_(1*−y−n*)_)B1*_x_* (B2*_m_*B3_(1*−x−m*)_)O_3_, where *x* and *y* range from 0.6 to 1.0 in increments of 0.01, and 0 ≤ *n* ≤ 0.4, 0 ≤ *m* ≤ 0.4. This formula facilitates the creation of millions of ABO_3_-type perovskites. It is generally assumed that stable perovskites can form if the tolerance factor (***t_f_***) falls within the range of 0.8~1.0. The formula for ***t_f_*** is as follows:

(1) $$t_{f}=\frac{R_{A}{+R}_{O}}{\sqrt{2}{(R}_{B}{+R}_{O})}$$

where $R_{A}$, $R_{B}$, and $R_{O}$ represent the ionic radii of A-site, B-site, and O, respectively.

The virtual samples were generated to screen perovskites with high SSA considering the elements present in the perovskites from the existing dataset and the conditions outlined above.

#### 2.1.6. Mechanism Mining

Mechanism mining is essential in materials research, particularly for understanding how the features affect the SSA of ABO_3_-type perovskites. The features that most significantly impact the SSA of ABO_3_-type perovskites can be identified by leveraging the interpretability of ML results. Mechanism mining can help to adjust the material design approach and provide precise guidance for material synthesis. The effectiveness of mechanism mining directly influences the practical application and usability of the models developed.

### 2.2. Computational Details

In this study, we conducted most of the ML calculations using HyperMiner software package (2009 edition) [50] and the in-house OCPMDM [51,52,53]. HyperMiner is available for free download from the website of our laboratory (http://materials-data-mining.com/home). Detailed instructions for using OCPMDM can be accessed from the webserver link (http://materials-data-mining.com/ocpmdm/).

## 3. Results and Discussion

To enhance the predictive performance of ML models, it is essential to consider different types of models and compare their predictive accuracy based on the dataset’s characteristics and the suitability of the algorithms. The SVR algorithm offers strict mathematical theoretical support, strong interpretability, and robustness. However, its training process requires significant computational resources and storage capacity, making it more suitable for smaller sample sets.

In this study, we used the widely used ML algorithms to establish the predictive models of SSA of ABO_3_-type perovskites. These algorithms include decision tree regression (DTR), gradient boosting regression (GBR), partial least squares (PLS), relevance vector machine (RVM), SVR-RBF, SVR with linear kernel function (SVR-LKF), SVR with polynomial kernel function (SVR-PKF), random forest regression (RFR), and back propagation neural network (BPNN). The root mean square error (RMSE) and Pearson correlation coefficient (R) of the LOOCV results of the nine algorithms are presented in Table 1. 

The model using the SVR-RBF algorithm was more effective for predicting the SSA of ABO_3_-type perovskites compared to the other algorithms. This conclusion is supported by its lowest RMSE and highest Pearson correlation coefficient (R) in the LOOCV results. SVR, which operates on the principle of structural risk minimization, addresses issues such as uncertain network structure, overfitting, underfitting, and local minima commonly encountered in some algorithms such as artificial neural networks [54]. It is widely considered as one of the best methods for small sample regression problems [55]. Leveraging slack variables and kernel functions, SVR is effective at handling situations where the data are linearly inseparable. RBF kernel is the most commonly used kernel function in SVR, known for its applicability to both small and large sample problems, as well as high-dimensional and low-dimensional datasets. This analysis shows that SVR-RBF is the most suitable for the dataset. Therefore, it was selected as the algorithm to construct the predictive model for the SSA of ABO_3_-type perovskites.

### 3.1. Feature Selection and Analysis

During the ML training process, datasets often contain many samples and features, some of which offer little or no value for modeling. When a significant proportion of the dataset comprises irrelevant features, it can prolong the model training time and increase the risk of underfitting [56]. Conversely, if a subset of data with minor impact account for a large proportion of the dataset, it can prolong the model training time and increase the risk of overfitting of the model [57]. Consequently, feature selection is a key step in building a reliable model [58]. The objective of feature selection is to identify the optimal subset of features from the original feature pool, one with essential information and less redundancy.

**Figure 3:**
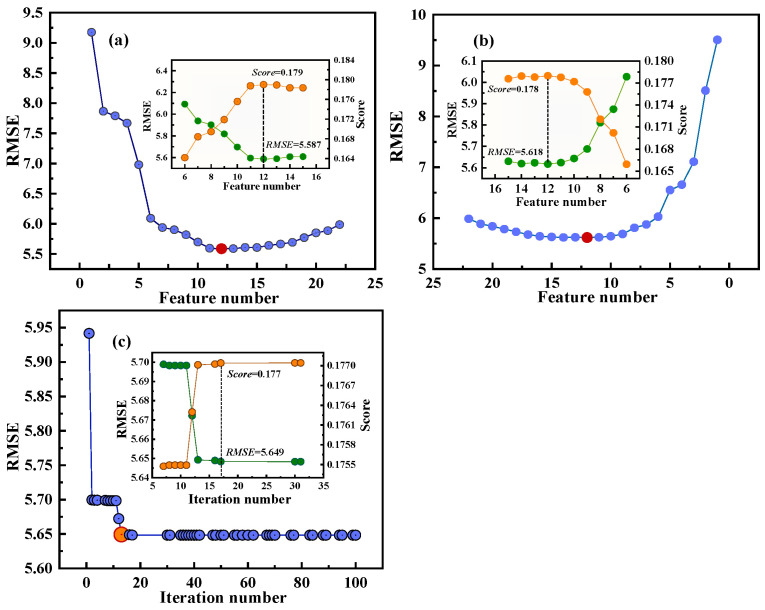
Feature screening using (**a**) FSM-SVR, (**b**) BSM-SVR, and (**c**) GA-SVR. The blue and red circles represent RMSE and the minimum RMSE, respectively. In the illustration, the green and orange circles represent RMSE and Score, respectively.

Three approaches based on SVR were used to identify the optimal subset. These approaches include forward selection method (FSM) [59], backward selection method (BSM) [60], and genetic algorithm (GA) [61,62]. FSM is a heuristic method that starts with an empty set and gradually adds features to the feature subsets based on importance. At each stage, a feature that maximizes the model performance is selected to train the ML model. This iterative process continues until the main features contributing significantly to the model are identified and retained for modeling. The principle of BSM is similar to FSM, except that all features are initially included in training the models. Subsequently, iteratively, the feature that contributes the least to model performance is removed and the remaining features are used to train the ML model. GA is a global optimization algorithm that simulates the evolutionary principles observed in biological systems, where only the fittest survives. The search processes of the three methods (illustrated in Figure 3) continue until a feature subset closely approaching the optimal solution is obtained. Notably, the RMSE value first decreases with an increase in the feature number and gradually increases after reaching a minimum value (Figure 3a,b). In addition, the RMSE initially decreases sharply with an increase in the iteration number (Figure 3c). The RMSE reaches its minimum value after 17 iterations and then stabilizes. Conversely, the parameter “score” exhibits an opposite trend to RMSE for the three methods (Figure 3). Comparison of the three methods showed that the FSM-SVR method was superior to the other algorithms, achieving the lowest RMSE and highest score. According to the results of the FSM-SVR method, the optimal subset comprised 12 variables: ***CT***, ***AT***, ***DT***, ***PM***, ***Z_IA_***, **Δ*fus_A_***, ***T_mA_***, ***T_mB_***, ***T_bA_***, ***ρ_A_***, ***EA_a_***, and ***EA_b_***.

To further refine the dataset used for modeling, a new dataset was generated incorporating these 12 features and six principal components [63] (***P_CA_*_1_**, ***P_CA_*_2_**, ***P_CA_*_3_**, ***P_CA_*_4_**, ***P_CA_*_5_**, and ***P_CA_*_6_**) calculated based on the 12 features. The formulas of the principal components are presented as Equations (S1)~(S6) in the Supporting Information. Moreover, nine key features were identified using BSM-SVR method for modeling: ***CT***, ***DT***, ***PM, T_mB_***, ***ρ_A_***, ***EA_a_***, ***EA_b_***, ***P_CA_*_3_**, and ***P_CA_*_6_**.

### 3.2. Model Construction

To establish an effective model, it is not only essential to select appropriate algorithms and key features for modeling but also to optimize the hyperparameters of the algorithms and evaluate the models using appropriate methods [64].

#### 3.2.1. Optimizing Hyperparameters

**Figure 4:**
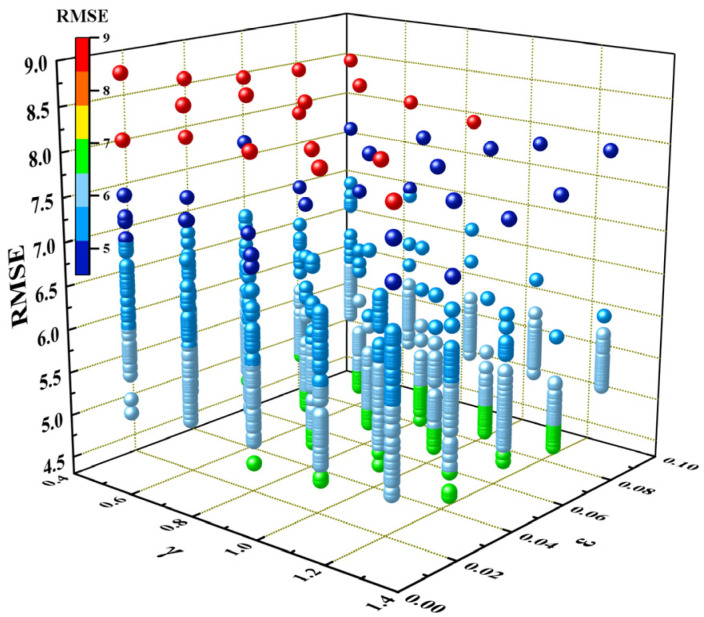
RMSE versus γ and ε obtained in the optimization process of the algorithm hyperparameters.

Hyperparameter optimization [65] involves finding the optimal combination of hyperparameters to maximize model performance. Therefore, the three hyperparameters [54] (namely, ε, C, and γ) of SVR-RBF must be adjusted to enhance accuracy and generalization ability of the SVR-RBF model. The parameter ε controls the smoothness of regression curves, influencing the model’s tolerance for errors. C is a regularization constant that controls the penalty intensity applied to the model based on errors encountered during training. This parameter determines the trade-off between errors and complexity of the model. The parameter γ is a crucial hyperparameter that controls the kernel function, determining the impacts of sample points on the model. The ranges of ε, C, and γ used to optimize the model are (0.01, 0.09), (1, 100), and (0.5, 1.5), and the corresponding step sizes are 0.02, 2, and 0.1, respectively. The optimization process involved using the grid search method with 10-fold cross-validation to find the optimal combination of these hyperparameters. The search process is illustrated in Figure 4. The optimal values of ε, C, and γ were 0.07, 97, and 0.9, resulting in a minimal RMSE value of 4.584, respectively.

#### 3.2.2. Establishing Model

The SVR-RBF model was constructed using the key features and the optimal hyperparameters. The expression of the model is shown below:

(2) $$\mathrm{SSA}=\overset{n}{\underset{i=1}{\sum }}\beta_{i}\exp\left((-0.9)\;\times \;{\left|\left|X-X_{i}\right|\right|}^{2}\right)-0.0927$$

where *n* and *β_i_* represent the corresponding number and Lagrange multiplier of the support vectors, respectively. *X* and *X_i_* denote the unknown vector and the support vector, respectively.

**Figure 5:**
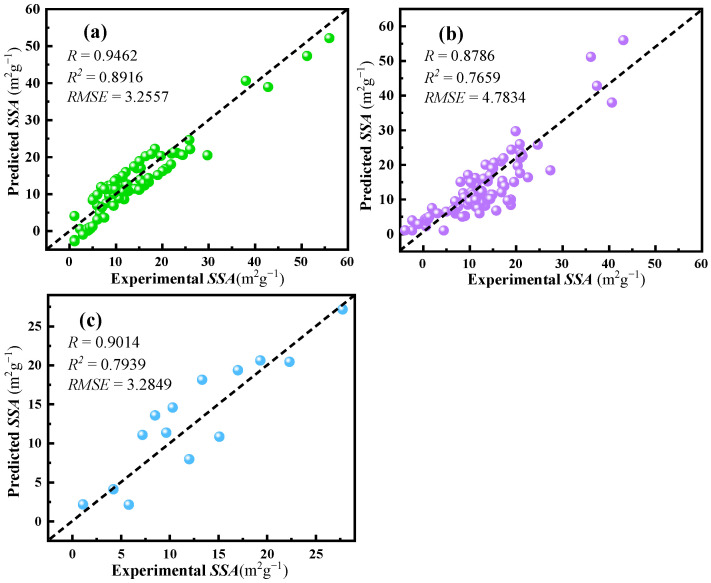
The experimental versus predicted SSA of the samples in (**a**) the training set; (**b**) LOOCV; (**c**) the testing set.

R, the coefficient of determination (R^2^), and RMSE were indices to evaluate the performance of the SVR-RBF model by comparing the experimental and predicted SSA values of ABO_3_-type perovskites. Figure 5a illustrates the experimental SSA versus the predicted SSA for the samples in the training set. The SVR-RBF model demonstrated relatively high accuracy, with R, R^2^, and RMSE values of 0.9462, 0.8916, and 3.2557, respectively.

#### 3.2.3. Model Evaluation

LOOCV, the testing set, and the validation set were used to verify the predictive performance of the ML model, with results shown in Figure 5b, Figure 5c, and Table 2, respectively. The model exhibited higher R and lower RMSE for LOOCV and the external set, indicating its effectiveness and reliability. The maximum relative error of the samples was +0.215, with very low relative errors of the other two samples, indicating that the ML model had robust predictive performance (Table 2). The higher relative prediction error observed for sample 101 can be attributed to the absence of the molecular formula of Gd in the samples in the training set, which is the element at the A-site of sample 101. The prediction range of the model can be expanded by collecting more training samples of ABO_3_-type perovskites containing Gd or other elements. In addition, the SVR model underwent 100 random 10-fold cross-validations using the training samples. The average R, R^2^, and RMSE values of the 10-fold cross-validations were 0.8695, 0.7538, and 4.8979, respectively. These results further demonstrate the robustness and generalization capability of the SVR-RBF model.

### 3.3. Model Application

Despite construction of several effective ML models across various fields [66], most of them are not readily accessible to experimental researchers. We adopted two strategies (developing an online web service and conducting virtual screening) to facilitate the utilization of these models by scientists conducting material experiments.

#### 3.3.1. Online Web Service

**Figure 6:**
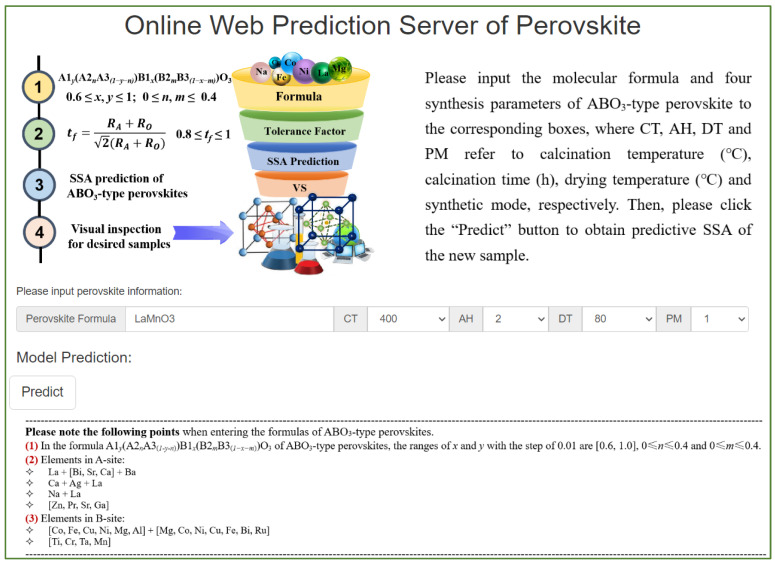
A screenshot of the web service for predicting SSA of ABO_3_-type perovskites.

We established a webserver based on the SVR-RBF model to simplify the prediction of SSA for ABO_3_-type perovskites, aiding in designing materials with desired properties. This webserver offers efficiency, convenience, and flexibility in guiding the design and development of ABO_3_-type perovskites. A screenshot of the webserver (http://1.14.49.110/online_predict/Perovskite accessed on 7 June 2024) is shown in Figure 6. To use the webserver, users input the chemical formulas of ABO_3_-type perovskites into the corresponding dialog box and select values for the four synthetic parameters need be selected from dropdown lists. The guidelines for inputting the molecular formulas of perovskites are provided in small characters at the bottom of Figure 6. The OCPMDM tool automatically generates the atomic parameters based on the formulas provided, eliminating the need for users to enter them manually. Subsequently, the predictive value of SSA of the sample is generated by clicking the “Predict” button. This entire predictive process typically takes a few seconds. 

#### 3.3.2. Virtual Screening

**Figure 7:**
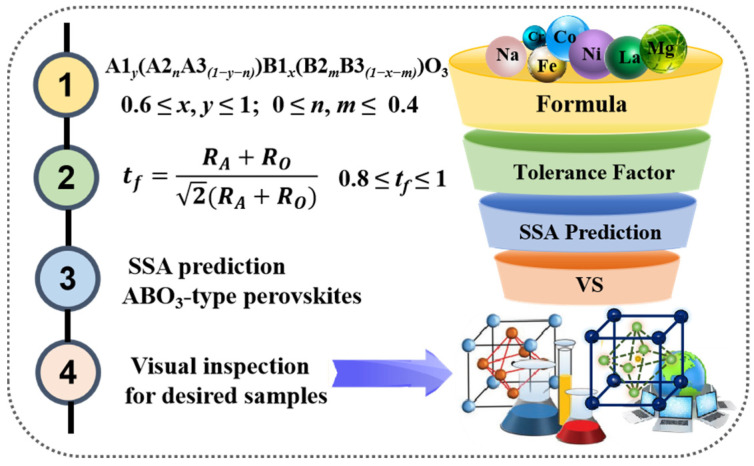
Virtual screening of ABO_3_-type perovskites.

The results based on the LOOCV and external test indicated the effectiveness and reliability of the SVR-RBF model in predicting the SSA of ABO_3_-type perovskites. Consequently, the model was used to predict the SSA of virtual samples to identify potential candidates. A schematic representation of the virtual screening of ABO_3_-type perovskites is shown in Figure 7. 

The virtual screening process is summarized below:

(1) In the formula A1*_y_*(A2*_n_*A3_(1*−y−n*)_)B1*_x_*(B2*_m_*B3_(1*−x−m*)_)O_3_ for ABO_3_-type perovskites, the values of *x* and *y* range from 0.6 to 1.0 in increments of 0.01, with 0 ≤ *n* ≤ 0.4 and 0 ≤ *m* ≤ 0.4.

(2) The tolerance factor ***t_f_*** of perovskites ranged from 0.8 to 1.0 to ensure a stable perovskite structure.

(3) The A-site and B-site allows for a maximum of three and two different doping ions, respectively.

(4) When the first element in the A-site space is La, the second element can be Bi, Sr, or Ca with a doping ratio ranging from 0.0 to 0.4. The third element in the A-site space is Ba with the remaining doping ratio.

(5) When the first element in the A-site space is Ca, the second element in A-site space is Ag and the third element is La with the remaining doping ratio.

(6) When the first element in the A-site space is Na, the second element is La with the remainder of the doping ratio.

(7) The elements in A-site space can be Zn, Pr, Sr, or Ga without doping other elements.

(8) When the first element in the B-site is Co, Fe, Cu, Ni, Mg, or Al, the second element can be Mg, Co, Ni, Cu, Fe, Bi, or Ru, with the remaining doping ratio, ensuring that the second element is different from the first element.

(9) The element in the B-site can be Ti, Cr, Ta, or Mn without doping other elements.

(10) The ranges of ***CT***, ***AT***, and ***DT*** are (400 °C, 1000 °C), (2h, 15h), and (80 °C, 300 °C), respectively, with steps of 50 °C, 1 h, and 10 °C, respectively.

(11) The values of ***PM*** can be 1, 2, or 3.

We identified the samples with potentially higher SSA than the highest SSA (56 m^2^g^−1^) observed in the existing dataset through virtual screening and predictive calculations using the SVR-RBF model. The top two candidates are presented in Table 3, with the highest predictive SSA value reaching 67.884 m^2^g^−1^. 

### 3.4. Mechanism Mining

We evaluated the relationships between the key features of the model and SSA to further explore the synthesis mechanism of ABO_3_-type perovskites. The optimal conditions for the samples with higher SSA were identified by pattern recognition.

#### 3.4.1. Relationships between Key Features and SSA

**Figure 8:**
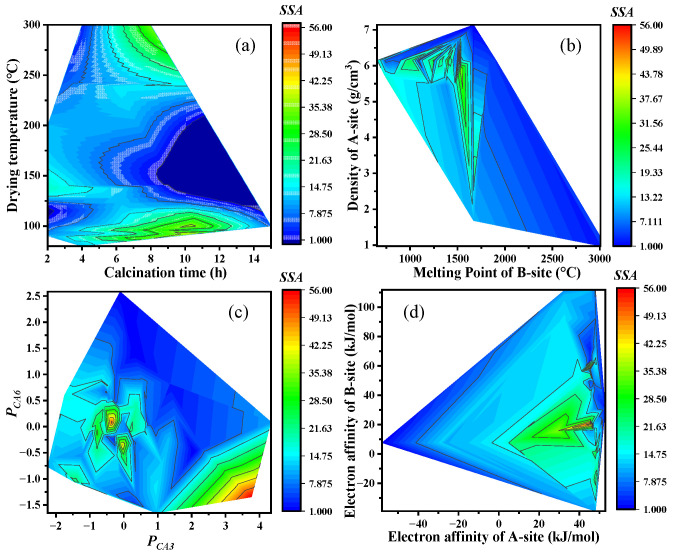
The relationships between SSA and the key features. (**a**) Calcination time and Drying temperature; (**b**) Melting point of B-site and Density of A-site; (**c**) ***P_CA_*_3_** and ***P_CA_*_6_**; (**d**) Electron affinity of A-site and Electron affinity of B-site.

The relationships between the key features and SSA are explored, and the findings are presented in Figure 8. The results indicate that ABO_3_-type perovskites may exhibit higher SSA when the drying temperature is either below 120 °C or above 250 °C (Figure 8a). Excessive calcination time tends to result in low SSA. In addition, the excessive melting point of B-site was not conducive to achieving a high SSA (Figure 8b). Conversely, a higher density of the A-site correlated with a higher SSA. Furthermore, a higher *P_CA_*_3_ value and a lower *P_CA_*_6_ value were associated with higher SSA (Figure 8c). A larger electron affinity at the A-site and a B-site electron affinity ranging between 0 and 40 kJ/mol potentially generated higher SSA (Figure 8d).

#### 3.4.2. Pattern Recognition

**Figure 9:**
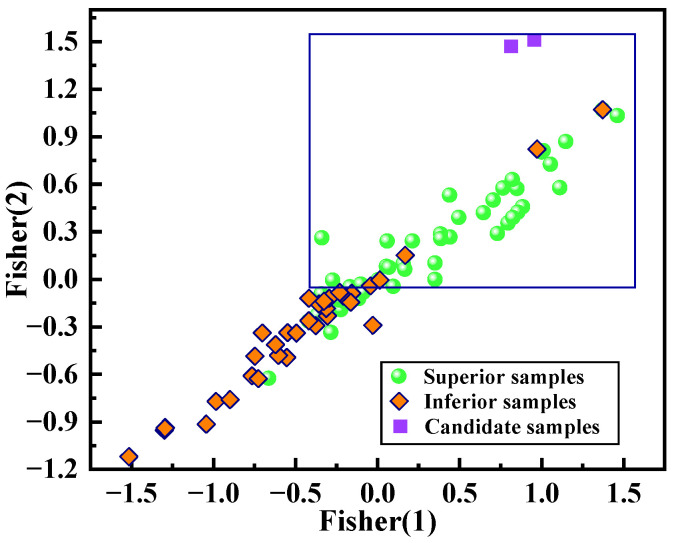
The projection of pattern recognition.

The samples were divided into two groups based on their SSA: “superior” with higher ***SSA*** and “inferior” with lower ***SSA***. The samples frequently have relatively high ***SSA*** in the superior region of the projection diagram. The projection of pattern recognition was obtained through the Fisher discriminant analysis method [67,68] and is illustrated in Figure 9. A clear distribution pattern was observed for the two categories of samples (Figure 9). The results showed that most of the superior samples were clustered in a rectangular area of the projection graph, accounting for approximately 90.24% of the total samples in that region. This indicates a significant concentration of samples with higher SSA in a specific area of the projection. The results of pattern recognition for all samples in the training set are summarized in Table 4. The two categories of samples exhibited F1_score equal to or exceeding 0.8, indicating robust performance of the pattern recognition model.

The top two samples with potentially higher SSA are highlighted by purple rectangles in Figure 9. The samples should fall within this rectangular region to achieve new ABO_3_-type perovskites with optimal SSA. The expressions defining the superior region are as follows:

(3) $$-0.4618\;\leq \;\mathrm{Fisher}\left(1\right)\;\leq \;1.565$$

(4) $$-0.0858\;\leq \;\mathrm{Fisher}\left(2\right)\;\leq \;1.587$$

## 4. Conclusions

The aim of this study was to construct an SVR model for accelerating the design of ABO_3_-type perovskites with optimal SSA, using a six-step approach. The analysis mainly focused on determining the influence of the key features on the SSA (1.05~56 m^2^g^−1^) of ABO_3_-type perovskites synthesized through the sol–gel technique. Results from LOOCV, external test, and the validation set demonstrate that the SVR-RBF model effectively predicts SSA of ABO_3_-type perovskites with robust accuracy. Key features affecting SSA of ABO_3_-type perovskites were identified using FSM and BSM incorporated in SVR-RBF. The perovskite candidates with higher SSA (67.884 m^2^g^−1^) than the highest value (56 m^2^g^−1^) of SSA observed using the existing dataset were identified by the SVR-RBF model, virtual screening, and pattern recognition. A webserver (http://1.14.49.110/online_predict/Perovskite (accessed on 7 June 2024)) based on the constructed model was developed to facilitate ease of use for researchers interested in ABO_3_-type perovskites. This webserver allows rapid and convenient prediction of SSA of ABO_3_-type perovskites and it is freely accessible. Furthermore, with the increase in relevant experimental data, we aim to collect more data and apply more algorithms to further enhance the predictive performance of the model in future endeavors. The approach outlined in this study is instrumental in accelerating material design and can be used for various applications in addition to ABO_3_-type perovskite materials.

## Figures and Tables

**Table 1:** The LOOCV results of the nine algorithms.

Index	DTR	GBR	PLS	RVM	SVR-RBF	SVR-LKF	SVR-PKF	RFR	BPNN
RMSE	7.570	5.798	8.588	6.304	4.895	8.408	7.286	7.618	10.301
R	0.688	0.817	0.504	0.791	0.870	0.540	0.679	0.640	0.443

**Table 2:** The predictive results based on the validation set.

No.	Molecular Formula	Experimental *SSA* (m^2^g^−1^)	Predictive *SSA* (m^2^g^−1^)	Relative Error
100^#^	LaFeO_3_	7	6.516	−0.0691
101^#^	GdCoO_3_	8.69	10.558	+0.215
102^#^	LaMnO_3_	25	23.830	−0.047

**Table 3:** The top two candidates with potentially higher SSA than the dataset value.

No.	Molecular Formula	*SSA* (m^2^g^−1^)	*DT* (°C)	*AH* (h)	*PM*	Fisher (1)	Fisher (2)
1	La_0.61_Ba_0.39_TiO_3_	67.884	280	9	1	0.953	1.511
2	La_0.51_Ba_0.49_TiO_3_	66.158	280	9	1	0.813	1.471

**Table 4:** The evaluation results of pattern recognition.

Index	“Superior”	“Inferior”
True Positives	37	32
False Positives	4	12
False Negatives	12	4
Precision	0.902	0.727
Recall	0.755	0.889
F1_score	0.822	0.8

## Data Availability

The original contributions presented in the study are included in the article/Supplementary Materials, further inquiries can be directed to the corresponding authors.

## Supplementary Materials

The following supporting information can be downloaded at: https://www.mdpi.com/article/10.3390/ma17123026/s1, Table S1: Overview of ML applications in ABO_3_-type perovskites; Table S2: The dataset including 102 ABO_3_-type perovskite samples; Table S3: The meanings of the twenty-five candidate features; Figure S1: Pearson correlation matrix diagram of the twenty-five features; Figure S2 The mRMR scores of the twenty-five features; Formulas for 6 principal components. References [37,38,69,70,71,72,73,74,75,76,77,78,79,80,81,82,83,84,85,86,87,88,89,90,91,92,93,94,95,96,97,98,99,100,101,102,103,104,105,106,107,108,109,110,111,112,113,114,115,116,117,118,119,120] are cited in the Supplementary Materials.

## Abbreviations

Abbreviation	Full Name
SSA	Specific Surface Area
ML	Machine Learning
SVR	Support Vector Regression
LOOCV	Leave-one-out Cross-validation
RBF	Radial Basis Function
***PM***	Synthetic Mode
***CT***	Calcination Temperature
***AH***	Calcination Time
***DT***	Drying Temperature
OCPMDM	Online Computational Platform of Material Data Mining
mRMR	Max Relevance Min Redundancy
***R_A_/R_B_***	Ratio of ionic radius
***α***	Unit cell lattice edge
***t_f_***	Tolerance factor
***R_A_***	Ionic Radius of A-site
***R_B_***	Ionic Radius of B-site
***R_O_***	Ionic Radius of O
DTR	Decision Tree Regression
GBR	Gradient Boosting Regression
PLS	Partial Least Squares
RVM	Relevance Vector Machine
LKF	Linear Kernel Function
PKF	Polynomial Kernel Function
RFR	Random Forest Regression
BPNN	Back Propagation Neural Network
RMSE	Root Mean Square Error
R	Pearson correlation coefficient
FSM	Forward Selection Method
BSM	Backward Selection Method
GA	Genetic Algorithm
***Z_IA_***	Ionization Energy of A-site
**Δ*fus_A_***	Enthalpy of fusion at the melting point of A-site
***T_mA_***	Melting Point of A-site
***T_mB_***	Melting Point of B-site
***T_bA_***	Boiling Point of A-site
***ρ_A_***	Density of A-site
***EA_a_***	Electron Affinity of A-site
***EA_b_***	Electron Affinity of B-site
***P_CA_*_3_**	Third principal component
***P_CA_*_6_**	Sixth principal component
R^2^	The coefficient of determination
