# Supplementary material for: Accelerated Design for Perovskite-Oxide-Based Photocatalysts Using Machine Learning Techniques
Source: Materials (Basel). 2024 Jun 20;17(12):3026. doi: 10.3390/ma17123026 (PMC11206125; doi:10.3390/ma17123026)
Supplement: Supplementary file 1 [file materials-17-03026-s001.zip › materials-2952500-supplementary.pdf]

**Supplementary Information**

# Accelerated Design for Perovskite-Oxide-Based Photocatalysts Using Machine Learning Techniques

**Xiuyun Zhai [1,*] and Mingtong Chen [2,*]**

1 College of Intelligent Manufacturing, Hunan University of Science and Engineering, Yongzhou 425199, China
2 Public Experimental Teaching Center, Panzhihua University, Panzhihua 617000, China
* Correspondence: cqfbb2008@shu.edu.cn (X.Z.); cmt2711@126.com (M.C.)

**Table S1** Overview of ML applications in $ABO_3$-type perovskites

| No. | Target properties | Algorithms | Ref |
|---|---|---|---|
| 1 | Thermodynamic phase stability | ET and KRR | [1] |
| 2 | Stable and metastable | GBDT | [2] |
| 3 | Formability and cubic structure stability | RF and Gradient boosting tree | [3] |
| 4 | Formation energy and bandgap | GBR | [4] |
| 5 | Curie temperature | SVR | [5] |
| 6 | Curie temperature | RR, SVM and ERT | [6] |
| 7 | Néel temperature | SVM | [7] |
| 8 | Maximum magnetic entropy change | GPR | [8] |
| 9 | Ionic conductivity | SVM | [9] |
| 10 | Specific surface area | SVM | [10] |
| 11 | The nature of band gap | RF | [11] |
| 12 | Thermodynamic stable | PSO-SVR and SVR | [12] |
| 13 | Crystal structure | Boosting algorithms, KNN and XGBoost | [13] |
| 14 | Perovskite structures | Quantum Machine Learning | [14] |
| 15 | Formability of perovskite structures | GBDT | [15] |
| 16 | Formation energy | DTR, GBRT, RFR and ETR | [16] |
| 17 | Defect formation energy | RFR | [17] |
| 18 | Thermal expansion coefficient | SVM | [18] |
| 19 | Oxygen ionic conductivities | GPR | [19] |
| 20 | Formability and stability | RF | [20] |
| 21 | Experimental design | ANN, Gaussian process and SVR | [21] |
| 22 | Stability | PCA and LFA | [22] |
| 23 | Band gaps and Band-edge positions | GBDT, LRR and KRR | [23] |
| 24 | Hydrogen production rate | GBR, BPANN and SVR | [24] |

**Table S2** The dataset including 102 $ABO_3$-type perovskite samples

| No. | Molecular formulas | ***SSA*** ($m^2g^{-1}$) | ***CT*** (°C) | ***AH*** (h) | *DT* (°C) | PM | Ref. |
|---|---|---|---|---|---|---|---|
| 1 | $ZnTiO_3$ | 1.05 | 900 | 2 | 120 | 1 | [25] |
| 2 | $LaFeO_3$ | 1.08 | 900 | 4 | 150 | 3 | [26] |
| 3 | $LaCo_{0.94}Mg_{0.06}O_3$ | 19 | 750 | 4 | 110 | 3 | [27] |
| 4 | $LaCo_{0.90}Mg_{0.1}0O_3$ | 21 | 750 | 4 | 110 | 3 | [27] |
| 5 | $LaCo_{0.80}Mg_{0.2}0O_3$ | 22 | 750 | 4 | 110 | 3 | [27] |
| 6 | $La_{0.5}Bi_{0.2}Ba_{0.2}Mn_{0.1}FeO_3$ | 20.63 | 600 | 4 | 120 | 3 | [28] |
| 7 | $La_{0.5}Bi_{0.2}Ba_{0.2}Mn_{0.1}FeO_3$ | 12.46 | 700 | 4 | 120 | 3 | [28] |
| 8 | $La_{0.5}Bi_{0.2}Ba_{0.2}Mn_{0.1}FeO_3$ | 5.91 | 800 | 4 | 120 | 3 | [28] |
| 9 | $La_{0.5}Bi_{0.2}Ba_{0.2}Mn_{0.1}FeO_3$ | 4.19 | 900 | 4 | 120 | 3 | [28] |
| 10 | $LaCrO_3$ | 3.95 | 600 | 5 | 100 | 3 | [29] |
| 11 | $LaMg_{0.2}Cr_{0.8}O_3$ | 8.42 | 600 | 5 | 100 | 3 | [29] |
| 12 | $LaMg_{0.4}Cr_{0.6}O_3$ | 29.71 | 600 | 5 | 100 | 3 | [29] |
| 13 | $LaMg_{0.6}Cr_{0.4}O_3$ | 18.41 | 600 | 5 | 100 | 3 | [29] |
| 14 | $LaMg_{0.8}Cr_{0.2}O_3$ | 14.46 | 600 | 5 | 100 | 3 | [29] |
| 15 | $PrFeO_3$ | 10.88 | 700 | 5 | 90 | 3 | [30] |
| 16 | $LaFe_{0.9}Co_{0.1}O_3$ | 51.2 | 750 | 10 | 110 | 3 | [31] |
| 17 | $LaFe_{0.1}Co_{0.9}O_3$ | 42.8 | 750 | 10 | 110 | 3 | [31] |
| 18 | $SrTiO_3$ | 16.4 | 650 | 10 | 110 | 3 | [32] |
| 19 | $La_{0.002}Sr_{0.998}TiO_3$ | 19.7 | 650 | 10 | 110 | 3 | [32] |
| 20 | $La_{0.01}Sr_{0.99}TiO_3$ | 24.1 | 650 | 10 | 110 | 3 | [32] |
| 21 | $La_{0.02}Sr_{0.98}TiO_3$ | 23.2 | 650 | 10 | 110 | 3 | [32] |
| 22 | $LaNiO_3$ | 14.1 | 600 | 2 | 130 | 2 | [33] |
| 23 | $LaNiO_3$ | 12.7 | 700 | 2 | 130 | 2 | [33] |
| 24 | $LaNiO_3$ | 11.8 | 800 | 2 | 130 | 2 | [33] |
| 25 | $LaNiO_3$ | 6.5 | 900 | 2 | 130 | 2 | [33] |
| 26 | $LaNiO_3$ | 12.2 | 600 | 6 | 130 | 2 | [33] |
| 27 | $LaFeO_3$ | 21.9 | 500 | 4 | 120 | 3 | [34] |
| 28 | $LaFeO_3$ | 15.37 | 600 | 4 | 120 | 3 | [34] |
| 29 | $LaFeO_3$ | 10.07 | 700 | 4 | 120 | 3 | [34] |
| 30 | $LaFeO_3$ | 5.24 | 800 | 4 | 120 | 3 | [34] |
| 31 | $SrTiO_3$ | 12.24 | 700 | 4 | 80 | 1 | [35] |
| 32 | $SrTiO_3$ | 10 | 700 | 4 | 80 | 1 | [36] |
| 33 | $LaCuO_3$ | 5 | 800 | 15 | 100 | 3 | [37] |
| 34 | $LaCu_{0.53}Ni_{0.47}O_3$ | 8.2 | 800 | 15 | 100 | 3 | [37] |
| 35 | $La_{0.8}Sr_{0.2}Ni_{0.8}Cu_{0.2}O_3$ | 9 | 700 | 5 | 100 | 3 | [38] |
| 36 | $La_{0.8}Sr_{0.2}Ni_{0.8}Fe_{0.2}O_3$ | 13.9 | 700 | 5 | 100 | 3 | [38] |
| 37 | $La_{0.8}Sr_{0.2}Ni_{0.8}Bi_{0.2}O_3$ | 12.1 | 700 | 5 | 100 | 3 | [38] |
| 38 | $LaNiO_3$ | 7.5 | 750 | 5 | 100 | 3 | [39] |
| 39 | $LaNi_{0.8}Fe_{0.2}O_3$ | 7.9 | 750 | 5 | 100 | 3 | [39] |
| 40 | $LaNi_{0.6}Fe_{0.4}O_3$ | 9.6 | 750 | 5 | 100 | 3 | [39] |
| 41 | $LaNi_{0.3}Fe_{0.7}O_3$ | 6.8 | 750 | 5 | 100 | 3 | [39] |
| 42 | $LaFeO_3$ | 15.1 | 750 | 5 | 100 | 3 | [39] |
| 43 | $LaFeO_3$ | 25.8 | 500 | 2 | 130 | 2 | [40] |
| 44 | $LaFeO_3$ | 22.55 | 600 | 2 | 130 | 2 | [40] |

| No. | Molecular formulas | ***SSA*** ($m^2g^{-1}$) | ***CT*** (°C) | ***AH*** (h) | *DT* (°C) | PM | Ref. |
|---|---|---|---|---|---|---|---|
| 45 | $LaFeO_3$ | 20.04 | 700 | 2 | 130 | 2 | [40] |
| 46 | $LaFeO_3$ | 8.5 | 800 | 2 | 130 | 2 | [40] |
| 47 | $GaFeO_3$ | 56 | 600 | 8 | 300 | 1 | [41] |
| 48 | $GaFeO_3$ | 38 | 700 | 8 | 300 | 1 | [41] |
| 49 | $GaFeO_3$ | 10 | 900 | 8 | 300 | 1 | [41] |
| 50 | $NaTaO_3$ | 2.25 | 900 | 12 | 120 | 3 | [42] |
| 51 | $Na_{0.995}La_{0.005}TaO_3$ | 3.83 | 900 | 12 | 120 | 3 | [42] |
| 52 | $Na_{0.985}La_{0.015}TaO_3$ | 4.55 | 900 | 12 | 120 | 3 | [42] |
| 53 | $Na_{0.98}La_{0.02}TaO_3$ | 4.54 | 900 | 12 | 120 | 3 | [42] |
| 54 | $Na_{0.95}La_{0.05}TaO_3$ | 4.36 | 900 | 12 | 120 | 3 | [42] |
| 55 | $SrTiO_3$ | 16.2 | 900 | 4 | 80 | 3 | [43] |
| 56 | $Ca_{0.98}Ag_{0.01}La_{0.01}TiO_3$ | 10.61 | 850 | 10 | 120 | 3 | [44] |
| 57 | $Ca_{0.96}Ag_{0.02}La_{0.02}TiO_3$ | 11.39 | 850 | 10 | 120 | 3 | [44] |
| 58 | $Ca_{0.94}Ag_{0.03}La_{0.03}TiO_3$ | 12.17 | 850 | 10 | 120 | 3 | [44] |
| 59 | $Ca_{0.92}Ag_{0.04}La_{0.04}TiO_3$ | 11.44 | 850 | 10 | 120 | 3 | [44] |
| 60 | $LaFeO_3$ | 9.5 | 700 | 4 | 90 | 3 | [45] |
| 61 | $LaFeO_3$ | 11.39 | 600 | 5 | 150 | 3 | [46] |
| 62 | $LaMg_{0.2}Fe_{0.8}O_3$ | 15.07 | 600 | 5 | 150 | 3 | [46] |
| 63 | $LaMg_{0.4}Fe_{0.6}O_3$ | 17.63 | 600 | 5 | 150 | 3 | [46] |
| 64 | $LaMg_{0.6}Fe_{0.4}O_3$ | 24.41 | 600 | 5 | 150 | 3 | [46] |
| 65 | $LaMgO_3$ | 10.17 | 600 | 5 | 150 | 3 | [46] |
| 66 | $PrCo_{0.7}Fe_{0.3}O_3$ | 7.9 | 700 | 2 | 90 | 3 | [47] |
| 67 | $PrCo_{0.6}Fe_{0.4}O_3$ | 8.7 | 700 | 2 | 90 | 3 | [47] |
| 68 | $PrCo_{0.5}Fe_{0.5}O_3$ | 7.5 | 700 | 2 | 90 | 3 | [47] |
| 69 | $PrCo_{0.3}Fe_{0.7}O_3$ | 9.9 | 700 | 2 | 90 | 3 | [47] |
| 70 | $PrCo_{0.1}Fe_{0.9}O_3$ | 9.7 | 400 | 2 | 90 | 3 | [47] |
| 71 | $PrCo_{0.1}Fe_{0.9}O_3$ | 15.2 | 600 | 2 | 90 | 3 | [47] |
| 72 | $PrCo_{0.1}Fe_{0.9}O_3$ | 15 | 700 | 2 | 90 | 3 | [47] |
| 73 | $PrCo_{0.1}Fe_{0.9}O_3$ | 9.5 | 800 | 2 | 90 | 3 | [47] |
| 74 | $LaMnO_3$ | 26 | 600 | 4 | 120 | 3 | [48] |
| 75 | $LaFeO_3$ | 19 | 600 | 4 | 120 | 3 | [48] |
| 76 | $LaCoO_3$ | 16 | 600 | 4 | 120 | 3 | [48] |
| 77 | $LaCoO_3$ | 1.1 | 750 | 4 | 300 | 3 | [49] |
| 78 | $LaCo_{0.8}Ru_{0.2}O_3$ | 2.9 | 750 | 4 | 300 | 3 | [49] |
| 79 | $LaCo_{0.6}Ru_{0.4}O_3$ | 5 | 750 | 4 | 300 | 3 | [49] |
| 80 | $LaAlO_3$ | 17.1 | 800 | 2 | 200 | 3 | [50] |
| 81 | $LaAl_{0.9}Ni_{0.1}O_3$ | 6 | 800 | 2 | 200 | 3 | [50] |
| 82 | $LaAl_{0.8}Ni_{0.2}O_3$ | 7 | 800 | 2 | 200 | 3 | [50] |
| 83 | $LaAl_{0.7}Ni_{0.3}O_3$ | 6 | 800 | 2 | 200 | 3 | [50] |
| 84 | $La_{0.9}Ca_{0.1}Al_{0.9}Ni_{0.1}O_3$ | 17 | 800 | 2 | 200 | 3 | [50] |
| 85 | $La_{0.9}Ca_{0.1}Al_{0.8}Ni_{0.2}O_3$ | 15 | 800 | 2 | 200 | 3 | [50] |
| 86* | $LaCoO_3$ | 17 | 750 | 4 | 110 | 1 | [27] |
| 87* | $La_{0.5}Bi_{0.2}Ba_{0.2}Mn_{0.1}FeO_3$ | 27.75 | 500 | 4 | 120 | 1 | [28] |
| 88* | $LaFeO_3$ | 8.5 | 700 | 3 | 110 | 3 | [51] |
| 89* | $La_{0.005}Sr_{0.995}TiO_3$ | 22.3 | 650 | 10 | 110 | 3 | [32] |
| 90* | $LaNiO_3$ | 15.1 | 600 | 4 | 130 | 2 | [33] |

| No. | Molecular formulas | ***SSA*** ($m^2g^{-1}$) | ***CT*** (°C) | ***AH*** (h) | ***DT*** (°C) | PM | Ref. |
|---|---|---|---|---|---|---|---|
| 91* | $LaFeO_3$ | 1.09 | 1000 | 4 | 120 | 1 | [34] |
| 92* | $La_{0.8}Sr_{0.2}Ni_{0.8}Co_{0.2}O_3$ | 10.3 | 700 | 5 | 100 | 3 | [38] |
| 93* | $LaFeO_3$ | 5.8 | 900 | 2 | 130 | 2 | [40] |
| 94* | $Na_{0.92}La_{0.08}TaO_3$ | 4.21 | 900 | 12 | 120 | 1 | [42] |
| 95* | $CaTiO_3$ | 9.64 | 850 | 10 | 120 | 1 | [44] |
| 96* | $LaMg_{0.8}Fe_{0.2}O_3$ | 13.32 | 600 | 5 | 150 | 3 | [46] |
| 97* | $PrCo_{0.9}Fe_{0.1}O_3$ | 7.2 | 700 | 2 | 90 | 3 | [47] |
| 98* | $PrCo_{0.1}Fe_{0.9}O_3$ | 19.3 | 500 | 2 | 90 | 1 | [47] |
| 99* | $La_{0.9}Ca_{0.1}Al_{0.7}Ni_{0.3}O_3$ | 12 | 800 | 2 | 200 | 3 | [50] |
| 100[#] | $LaFeO_3$ | 7 | 850 | 4 | 110 | 3 | [52] |
| 101[#] | $GdCoO_3$ | 8.69 | 750 | 4 | 110 | 3 | [53] |
| 102[#] | $LaMnO_3$ | 25 | 700 | 3 | 110 | 3 | [54] |

**Table S3** The meanings of the twenty-five candidate features

| No. | Meanings | Features | No. | Meanings | Features |
|---|---|---|---|---|---|
| 1 | Calcination Temperature (°C) | ***CT*** | 14 | Unit Cell Lattice Edge | $\boldsymbol{\alpha}$ |
| 2 | Calcination Time (h) | ***AH*** | 15 | Enthalpy of fusion at the melting point of A-site (kJ/mol) | $\boldsymbol{\Delta fus_A}$ |
| 3 | Drying Temperature (°C) | ***DT*** | 16 | Enthalpy of fusion at the melting point of B-site (kJ/mol) | $\boldsymbol{\Delta fus_B}$ |
| 4 | Synthetic Mode | ***PM*** | 17 | Melting Point of A-site (°C) | $\boldsymbol{T_{mA}}$ |
| 5 | Ionic Radius of A-site (pm) | $\boldsymbol{R_A}$ | 18 | Melting Point of B-site (°C) | $\boldsymbol{T_{mB}}$ |
| 6 | Ionic Radius of B-site (pm) | $\boldsymbol{R_B}$ | 19 | Boiling Point of A-site (°C) | $\boldsymbol{T_{bA}}$ |
| 7 | Ratio of Ionic Radius | $\boldsymbol{R_A/R_B}$ | 20 | Boiling Point of B-site (°C) | $\boldsymbol{T_{bB}}$ |
| 8 | Tolerance Factor | $\boldsymbol{T_f}$ | 21 | Density of A-site (g/cm$^3$) | $\boldsymbol{\rho_A}$ |
| 9 | Molecular Mass (g/mol) | ***M*** | 22 | Density of B-site (g/cm$^3$) | $\boldsymbol{\rho_B}$ |
| 10 | Pauling Electronegativity of A-site | $\boldsymbol{\chi_{pA}}$ | 23 | Electron Affinity of A-site (kJ/mol) | $\boldsymbol{EA_a}$ |
| 11 | Pauling Electronegativity of B-site | $\boldsymbol{\chi_{pB}}$ | 24 | Electron Affinity of B-site (kJ/mol) | $\boldsymbol{EA_b}$ |
| 12 | Ionization Energy of A-site (eV) | $\boldsymbol{Z_{IA}}$ | 25 | Critical Radius | $\boldsymbol{r_c}$ |
| 13 | Ionization Energy of B-site (eV) | $\boldsymbol{Z_{IB}}$ | | | |

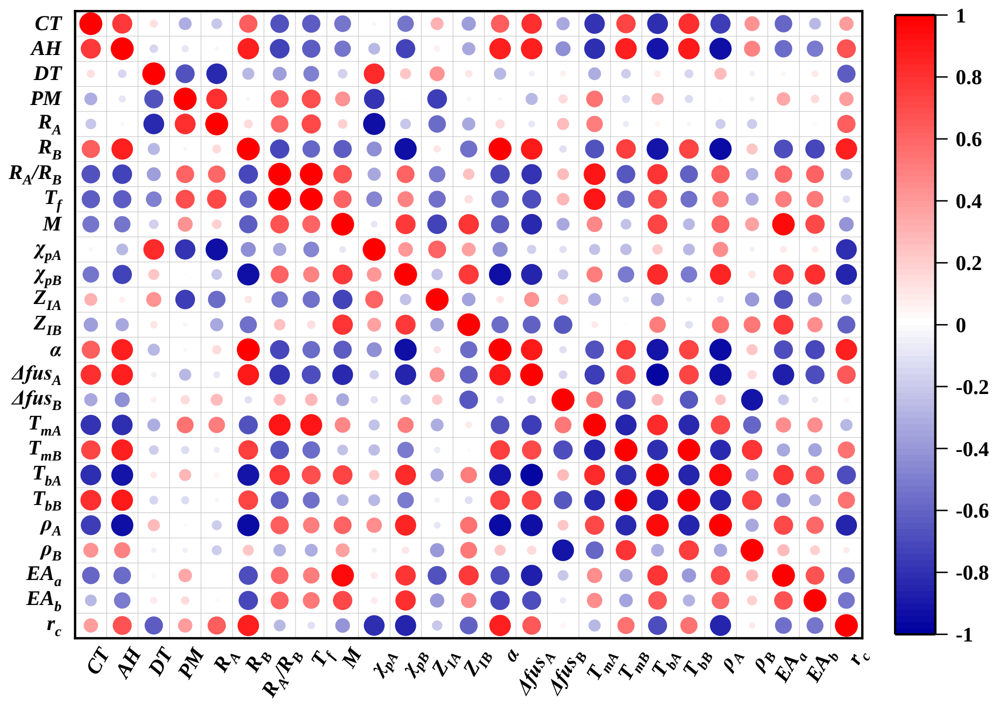


**Figure S1** Pearson correlation matrix diagram of the twenty-five features

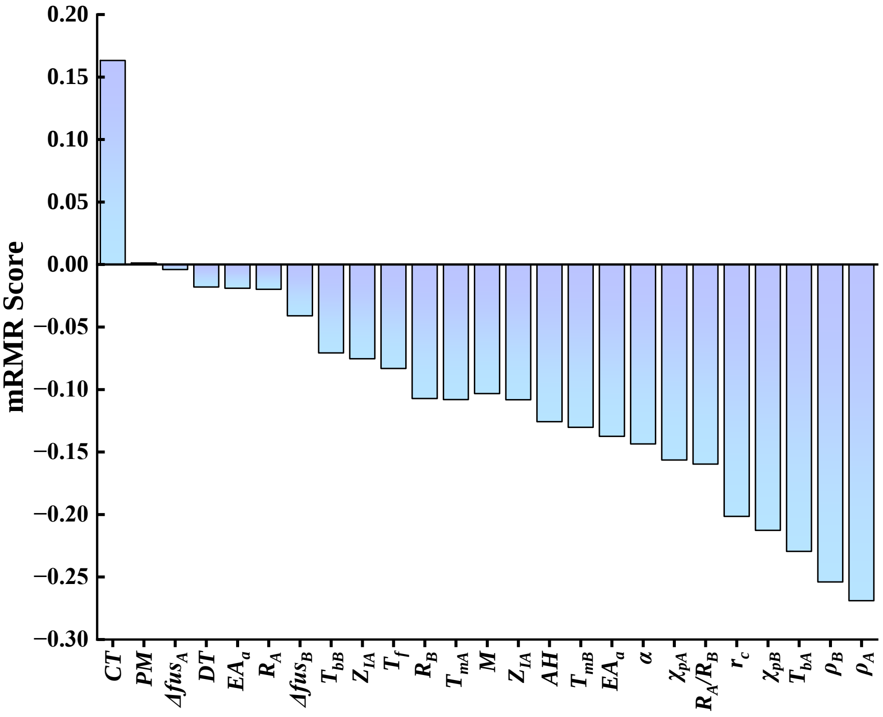


**Figure S2 The** mRMR scores of the twenty-five features

$$P_{CA1} = -0.259Z_{IA}+0.012EA_a+0.00365EA_b+0.00134T_{mA}-0.000692T_{mB}+0.000495T_{bA}-0.0105\Delta fus_A+0.2355\rho_A-0.00216CT-0.0934AH+5.448\text{E-}05DT+0.1657PM+0.3918 \tag{S1}$$

$$P_{CA2} = 1.252Z_{IA}-0.0176EA_a-0.00437EA_b+0.000174T_{mA}-0.000698T_{mB}-3.958\text{E-}05T_{bA}+0.00197\Delta fus_A+0.1162\rho_A-0.0004163CT-0.0859AH+0.003415DT-0.782PM-3.542 \tag{S2}$$

$$P_{CA3} = -0.1473Z_{IA}+0.01277EA_a+0.00852EA_b-0.001796T_{mA}+0.000290T_{mB}+3.97\text{E-}05T_{bA}-0.005132\Delta fus_A+0.07203\rho_A+0.00192CT+0.00353AH+0.01098DT-0.6485PM-0.1763 \tag{S3}$$

$$P_{CA4} = 0.3796Z_{IA}-0.006746EA_a+0.01845EA_b+0.001076T_{mA}-0.000336T_{mB}+6.914\text{E-}05T_{bA}+0.00348\Delta fus_A+0.003075\rho_A+0.00531CT+0.01077AH-0.001409DT+0.3009PM-7.915 \tag{S4}$$

$$P_{CA5} = 0.1268Z_{IA}-0.00104EA_a+0.0104EA_b-0.000318T_{mA}+0.0008359T_{mB}-7.796\text{E-}05T_{bA}-0.005807\Delta fus_A+0.01999\rho_A-0.001758CT-0.03819AH-0.01129DT-0.819PM+3.624 \tag{S5}$$

$$P_{CA6} = 0.591Z_{IA}+0.01055EA_a-0.01066EA_b-0.0006895T_{mA}+0.000652T_{mB}+1.383\text{E-}05T_{bA}-0.002944\Delta fus_A+0.1368\rho_A+0.004784CT-0.1168AH-0.00406DT+0.379PM-7.712 \tag{S6}$$

## References


[1] Li, W.; Jacobs R.; Morgan D. Predicting the thermodynamic stability of perovskite oxides using machine learning models. *Computational Materials Science* **2018**, 150, 454-463.

[2] Liu, H.Y.; Cheng J.C.; Dong H.Z.; Feng J.G.; Pang B.L.; Tian Z.Y.; Ma S.; Xia F.J.; Zhang C.K.; Dong L.F. Screening stable and metastable $ABO_3$ perovskites using machine learning and the materials project. *Computational Materials Science* **2020**, 177, 109614.

[3] Balachandran, P.V.; Emery A.A.; Gubernatis J.E.; Lookman T.; Wolverton C.; Zunger A. Predictions of new $ABO_3$ perovskite compounds by combining machine learning and density functional theory. *Physical Review Materials* **2018**, 2, 043802.

[4] Li, C.J.; Hao H.; Xu B.; Zhao G.H.; Chen L.H.; Zhang S.J.; Liu H.X. A progressive learning method for predicting the band gap of $ABO_3$ perovskites using an instrumental variable.

*Journal of Materials Chemistry C* **2020**, 8, 3127-3136.

[5] Zhai, X.Y.; Chen M.T.; Lu W.C. Accelerated search for perovskite materials with higher Curie temperature based on the machine learning methods. *Computational Materials Science* **2018**, 151, 41-48.

[6] Yang, Z.X.; Gao Z.R.; Sun X.F.; Cai H.L.; Zhang F.M.; Wu X.S. High critical transition temperature of lead-based perovskite ferroelectric crystals: A machine learning study. *Acta Physica Sinica* **2019**, 68, 210502.

[7] Xiao L.H., Z.Q., Xu X., Ji X.B. and Lu W.C. Support vector regression assisted predictions the néel temperature of perovskites manganites. *Computers and Applied Chemistry* **2018**, 35, 349-357.

[8] Zhang, Y.; Xu X.J. Machine learning the magnetocaloric effect in manganites from lattice parameters. *Applied Physics A-materials Science & Processing* **2020**, 126, 341.

[9] Xu, L.; Wencong L.; Chunrong P.; Qiang S.; Jin G. Two semi-empirical approaches for the prediction of oxide ionic conductivities in $ABO_3$ perovskites. *Computational Materials Science* **2009**, 46, 860-868.

[10] Shi, L.; Chang D.P.; Ji X.B.; Lu W.C. Using Data Mining To Search for Perovskite Materials with Higher Specific Surface Area. *Journal of Chemical Information and Modeling* **2018**, 58, 2420-2427.

[11] Priyanga, G.S.; Mattur M.N.; Nagappan N.; Rath S.; Thomas T. Prediction of nature of band gap of perovskite oxides ($ABO_3$) using a machine learning approach. *Journal of Materiomics* **2022**, 8, 937-948.

[12] Chen, L.P.; Wang X.C.; Xia W.J.; Liu C.H. PSO-SVR predicting for the Ehull of $ABO_3$-type compounds to screen the thermodynamic stable perovskite candidates based on multi-scale descriptors. *Computational Materials Science* **2022**, 211, 111435.

[13] Priyadarshini, R.; Joardar H.; Bisoy S.K.; Badapanda T. Crystal structural prediction of perovskite materials using machine learning: A comparative study. *Solid State Communications* **2023**, 361, 115062.

[14] Naseri, M.; Gusarov S.; Salahub D.R. Quantum Machine Learning in Materials Prediction: A Case Study on $ABO_3$ Perovskite Structures. *Journal of Physical Chemistry Letters* **2023**, 14, 6940-6947.

[15] Chen, L.P.; Xia W.J.; Yao T.Z. Identifying descriptors for perovskite structure of composite oxides and inferring formability via low-dimensional described features. *Computational Materials Science* **2023**, 226, 112216.

[16] Fan, X.Y. Prediction Of Formation Energy Using Two-Stage Machine Learning Based On Clustering. *Materiali In Tehnologije* **2021**, 55, 263-268.

[17] Sharma, V.; Kumar P.; Dev P.; Pilania G. Machine learning substitutional defect formation energies in $ABO_3$ perovskites. *Journal Of Applied Physics* **2020**, 128.

[18] McGuinness, K.P.; Oliynyk A.O.; Lee S.; Molero-Sanchez B.; Addo P.K. Machine-learning prediction of thermal expansion coefficient for perovskite oxides with experimental validation. *Physical Chemistry Chemical Physics* **2023**, 25, 32123-32131.

[19] Zhang, Y.; Xu X.J. Modeling oxygen ionic conductivities of $ABO_3$ Perovskites through machine learning. *Chemical Physics* **2022**, 558, 111511.

[20] Zhao, J.; Wang X.Y. Screening Perovskites from $ABO_3$ Combinations Generated by Constraint Satisfaction Techniques Using Machine Learning. *ACS Omega* **2022**, 7, 10483-

10491.
[21] Lourenço, M.P.; Tchagang A.; Shankar K.; Thangadurai V.; Salahub D.R. Active learning for optimum experimental design--insight into perovskite oxides. *Canadian Journal Of Chemistry* **2023**, 101, 734-744.
[22] Bhattacharya, S.; Roy A. Linking stability with molecular geometries of perovskites and lanthanide richness using machine learning methods. *Computational Materials Science* **2024**, 231, 112581.
[23] Li, W.; Wang Z.G.; Xiao X.; Zhang Z.Q.; Janotti A.; Rajasekaran S.; Medasani B. Predicting band gaps and band-edge positions of oxide perovskites using density functional theory and machine learning. *Physical Review B* **2022**, 106, 155156.
[24] Tao, Q.L.; Chang D.P.; Lu T.; Li L.; Chen H.M.; Yang X.; Liu X.J.; Li M.J.; Lu W.C. Multiobjective Stepwise Design Strategy-Assisted Design of High-Performance Perovskite Oxide Photocatalysts. *Journal of Physical Chemistry C* **2021**, 125, 21141-21150.
[25] Perween, S.; Ranjan A. Improved visible-light photocatalytic activity in $ZnTiO_3$ nanopowder prepared by sol-electrospinning. *Solar Energy Materials and Solar Cells* **2017**, 163, 148-156.
[26] Orak, C.; Atalay S.; Ersöz G. Photocatalytic and photo-Fenton-like degradation of methylparaben on monolith-supported perovskite-type catalysts. *Separation Science and Technology* **2017**, 52, 1310-1320.
[27] Sun, H.; Yang H.; Cui S.; Nie K.; Wu J. Simultaneous Mg-Modification Inside and Outside of $LaCoO_3$ Lattice and Their Photocatalytic Properties. *Chinese Journal of Inorganic Chemistry* **2016**, 32, 1704-1712.
[28] Abdulkadir, I.; Jonnalagadda S.B.; Martincigh B.S. Synthesis and effect of annealing temperature on the structural, magnetic and photocatalytic properties of $(La_{0.5}Bi_{0.2}Ba_{0.2}Mn_{0.1})FeO_{(3-\delta)}$. *Materials Chemistry and Physics* **2016**, 178, 196-203.
[29] Josephine, B.A.; Manikandan A.; Teresita V.M.; Antony S.A. Fundamental study of $LaMg_xCr_{1-x}O_{3-\delta}$ perovskites nano-photocatalysts: sol-gel synthesis, characterization and humidity sensing. *Korean Journal of Chemical Engineering* **2016**, 33, 1590-1598.
[30] Tijare, S.N.; Bakardjieva S.; Subrt J.; Joshi M.V.; Rayalu S.S.; Hishita S.; Labhsetwar N. Synthesis and visible light photocatalytic activity of nanocrystalline $PrFeO_3$ perovskite for hydrogen generation in ethanol-water system. *Journal of Chemical Sciences* **2014**, 126, 517-525.
[31] Tavakkoli, H.; Yazdanbakhsh M. Fabrication of two perovskite-type oxide nanoparticles as the new adsorbents in efficient removal of a pesticide from aqueous solutions: Kinetic, thermodynamic, and adsorption studies. *Microporous and Mesoporous Materials* **2013**, 176, 86-94.
[32] Li, H.; Cui Y.; Wu X.; Hong W.; Hua L. Effect of La contents on the structure and photocatalytic activity of La-$SrTiO_3$ catalysts. *Chinese Journal of Inorganic Chemistry*, 28, 2597-2604.
[33] Li, Y.; Yao S.; Wen W.; Xue L.; Yan Y. Sol–gel combustion synthesis and visible-light-driven photocatalytic property of perovskite $LaNiO_3$. *Journal of Alloys and Compounds* **2010**, 491, 560-564.
[34] Li, S.; Jing L.; Fu W.; Yang L.; Xin B.; Fu H. Photoinduced charge property of nanosized perovskite-type $LaFeO_3$ and its relationships with photocatalytic activity under visible

irradiation. *Materials Research Bulletin* **2007**, 42, 203-212.

[35] Puangpetch, T.; Sreethawong T.; Chavadej S. Hydrogen production over metal-loaded mesoporous-assembled $SrTiO_3$ nanocrystal photocatalysts: Effects of metal type and loading. *International Journal of Hydrogen Energy* **2010**, 35, 6531-6540.

[36] Puangpetch, T.; Sreethawong T.; Yoshikawa S.; Chavadej S. Hydrogen production from photocatalytic water splitting over mesoporous-assembled $SrTiO_3$ nanocrystal-based photocatalysts. *Journal of Molecular Catalysis A: Chemical* **2009**, 312, 97-106.

[37] Touahra, F.; Rabahi A.; Chebout R.; Boudjemaa A.; Lerari D.; Sehailia M.; Halliche D.; Bachari K. Enhanced catalytic behaviour of surface dispersed nickel on $LaCuO_3$ perovskite in the production of syngas: an expedient approach to carbon resistance during $CO_2$ reforming of methane. *International Journal of Hydrogen Energy* **2016**, 41, 2477-2486.

[38] Sutthiumporn, K.; Maneerung T.; Kathiraser Y.; Kawi S. $CO_2$ dry-reforming of methane over $La_{0.8}Sr_{0.2}Ni_{0.8}M_{0.2}O_3$ perovskite (M = Bi, Co, Cr, Cu, Fe): Roles of lattice oxygen on C–H activation and carbon suppression. *International Journal of Hydrogen Energy* **2012**, 37, 11195-11207.

[39] Luo, Y.; Wang X.; Qian Q.; Chen Q. Studies on B sites in Fe-doped $LaNiO_3$ perovskite for SCR of $NO_x$ with $H_2$. *International Journal of Hydrogen Energy* **2014**, 39, 15836-15843.

[40] Parida, K.M.; Reddy K.H.; Martha S.; Das D.P.; Biswal N. Fabrication of nanocrystalline $LaFeO_3$: An efficient sol–gel auto-combustion assisted visible light responsive photocatalyst for water decomposition. *International Journal of Hydrogen Energy* **2010**, 35, 12161-12168.

[41] Dhanasekaran, P.; Gupta N.M. Factors affecting the production of $H_2$ by water splitting over a novel visible-light-driven photocatalyst $GaFeO_3$. *International Journal of Hydrogen Energy* **2012**, 37, 4897-4907.

[42] Husin, H.; Chen H.-M.; Su W.-N.; Pan C.-J.; Chuang W.-T.; Sheu H.-S.; Hwang B.-J. Green fabrication of La-doped $NaTaO_3$ via $H_2O_2$ assisted sol–gel route for photocatalytic hydrogen production. *Applied Catalysis B: Environmental* **2011**, 102, 343-351.

[43] Bui, D.-N.; Mu J.; Wang L.; Kang S.-Z.; Li X. Preparation of Cu-loaded $SrTiO_3$ nanoparticles and their photocatalytic activity for hydrogen evolution from methanol aqueous solution. *Applied Surface Science* **2013**, 274, 328-333.

[44] Zhang, H.; Chen G.; He X.; Xu J. Electronic structure and photocatalytic properties of Ag–La codoped $CaTiO_3$. *Journal of Alloys and Compounds* **2012**, 516, 91-95.

[45] Tijare, S.N.; Joshi M.V.; Padole P.S.; Mangrulkar P.A.; Rayalu S.S.; Labhsetwar N.K. Photocatalytic hydrogen generation through water splitting on nano-crystalline $LaFeO_3$ perovskite. *International Journal of Hydrogen Energy* **2012**, 37, 10451-10456.

[46] Teresita, V.M.; Manikandan A.; Josephine B.A.; Sujatha S.; Antony S.A. Electromagnetic properties and humidity-sensing studies of magnetically recoverable $LaMg_xFe_{1-x}O_{3-\delta}$ perovskites nano-photocatalysts by Sol-Gel route. *Journal of Superconductivity and Novel Magnetism* **2016**, 29, 1691-1701.

[47] Sydorchuk, V.; Lutsyuk I.; Shved V.; Hreb V.; Kondyr A.; Zakutevskyy O.; Vasylechko L. $PrCo_{1-x}Fe_xO_3$ perovskite powders for possible photocatalytic applications. *Research on Chemical Intermediates* **2020**, 46, 1909-1930.

[48] Ibarra-Rodriguez, L.I.; Huerta-Flores A.M.; Mora-Hernandez J.M.; Torres-Martínez L.M. Photocatalytic evolution of $H_2$ over visible-light active $LaMO_3$(M: Co, Mn, Fe) perovskite

materials: Roles of oxygenated species in catalytic performance. *Journal of Physics and Chemistry of Solids* **2020**, 136, 109189.

[49] Mota, N.; Álvarez-Galván M.C.; Al-Zahrani S.M.; Navarro R.M.; Fierro J.L.G. Diesel fuel reforming over catalysts derived from $LaCo_{1-x}Ru_xO_3$ perovskites with high Ru loading. *International Journal of Hydrogen Energy* **2012**, 37, 7056-7066.

[50] Agüero, F.N.; Morales M.R.; Larrégola S.; Izurieta E.M.; Lopez E.; Cadús L.E. $La_{1-x}Ca_xAl_{1-y}NiyO_3$ perovskites used as precursors of nickel based catalysts for ethanol steam reforming. *International Journal of Hydrogen Energy* **2015**, 40, 15510-15520.

[51] Hu, R.; Li C.; Wang X.; Sun Y.; Jia H.; Su H.; Zhang Y. Photocatalytic activities of $LaFeO_3$ and $La_2FeTiO_6$ in p-chlorophenol degradation under visible light. *Catalysis Communications* **2012**, 29, 35-39.

[52] Boumaza, S.; Boudjellal L.; Brahimi R.; Belhadi A.; Trari M. Synthesis by citrates sol-gel method and characterization of the perovskite $LaFeO_3$: application to oxygen photo-production. *Journal of Sol-Gel Science and Technology* **2020**, 94, 486-492.

[53] Wang, J.; Li X.D.; Yu Z.Q.; Zhang S. Enhancing infrared emissivity of $GdCoO_3$ with Ca doping: Potential for advanced thermal control materials. *Ceramics International* **2024**, 50, 9630-9639.

[54] Guo, J.X.; Jing Y.; Shen T.; Luo H.D.; Liang J.; Yuan S.D. Effect of doped strontium on catalytic properties of $La_{1-x}Sr_xMnO_3$ for rhodamine B degradation. *Journal of Rare Earths* **2021**, 39, 1362-1369.
